# Microbial biosurfactant research: time to improve the rigour in the reporting of synthesis, functional characterization and process development

**DOI:** 10.1111/1751-7915.13704

**Published:** 2020-11-29

**Authors:** Matthew Simon Twigg, Niki Baccile, Ibrahim M. Banat, Eric Déziel, Roger Marchant, Sophie Roelants, Inge N. A. Van Bogaert

**Affiliations:** ^1^ School of Biomedical Sciences Ulster University Coleraine, Co. Londonderry BT52 1SA UK; ^2^ Centre National de la Recherche Scientifique Laboratoire de Chimie de la Matière Condensée de Paris Sorbonne Université LCMCP Paris F‐75005 France; ^3^ Centre Armand‐Frappier Santé Biotechnologie Institut National de la Recherche Scientifique (INRS) 531, Boul. Des Prairies Laval QC H7V 1B7 Canada; ^4^ Centre for Industrial Biotechnology and Biocatalysis (InBio.be) Faculty of Bioscience Engineering Ghent University Ghent Belgium; ^5^ Bio Base Europe Pilot Plant Rodenhuizenkaai 1 Ghent 9042 Belgium; ^6^ Centre for Synthetic Biology Department of Biotechnology Ghent University Coupure Links 653 Ghent 9000 Belgium

## Abstract

The demand for microbially produced surface‐active compounds for use in industrial processes and products is increasing. As such, there has been a comparable increase in the number of publications relating to the characterization of novel surface‐active compounds: novel producers of already characterized surface‐active compounds and production processes for the generation of these compounds. Leading researchers in the field have identified that many of these studies utilize techniques are not precise and accurate enough, so some published conclusions might not be justified. Such studies lacking robust experimental evidence generated by validated techniques and standard operating procedures are detrimental to the field of microbially produced surface‐active compound research. In this publication, we have critically reviewed a wide range of techniques utilized in the characterization of surface‐active compounds from microbial sources: identification of surface‐active compound producing microorganisms and functional testing of resultant surface‐active compounds. We have also reviewed the experimental evidence required for process development to take these compounds out of the laboratory and into industrial application. We devised this review as a guide to both researchers and the peer‐reviewed process to improve the stringency of future studies and publications within this field of science.

## Graphical abstract

The demand for microbially produced surface‐active compounds for use in industrial processes and products is increasing. As such there has been a comparable increase in the number of publications relating to the characterisation of novel surface‐active compounds; novel producers of already characterised surface‐active compounds and production processes for the generation of these compounds. We devised this review as a guide to both researchers and the peer review process to improve the stringency of future studies and publications within this field of science.

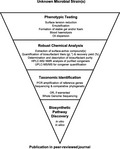

## Introduction

Surface‐active compounds (surfactants) have the ability to reduce tension at phase interfaces, emulsify oil in water and water in oil mixtures and aid in the formation of both stable gels and foams (Naughton *et al*., 2019). These amphiphilic compounds can be synthetically produced using precursors obtained from the petrochemical and/or oleochemical industry; however, microorganisms also synthesize different types of surfactants. These surface‐active compounds produced from biological sources such as bacteria, fungi and yeasts are termed either biosurfactants or bioemulsifiers (Marchant and Banat, 2012). The biological function of most of these compounds is not fully understood; however, among the proposed biological roles of these compounds are to facilitate the assimilation of poorly water‐soluble nutrients; serve as nutrient reserves; promote motility behaviours; aid in biofilm development; and act as antimicrobial and virulence factors (Kharazmi *et al*., 1989; Déziel et al., 1996; Haba, Pinazo, *et al*., 2003; Zulianello *et al*., 2006; Pamp and Tolker‐Nielsen, 2007; Tremblay *et al*., 2007; Alhede *et al*., 2009; Perfumo *et al*., 2010; Nickzad *et al*., 2015; De Clercq et al., 2020; Juma *et al*., 2020).

Microbial surface‐active compounds are classified based on their molecular structure. High molecular weight compounds, commonly designated as bioemulsifiers, include, lipoproteins, lipopolysaccharides, heteropolysaccharides and proteins. Low molecular weight compounds commonly referred to as biosurfactants include glycolipids (rhamnolipids, sophorolipids, mannosylerythritol lipids and trehalose lipids) and lipopeptides (e.g. surfactin, fengycin) (Rosenberg and Ron, 1999; Ongena *et al*., 2007; Van Bogaert *et al*., 2007; Morita *et al*., 2009; Abdel‐Mawgoud *et al*., 2010; Franzetti *et al*., 2010). Both high and low molecular weight surface‐active compounds can be utilized in a wide variety of applications including but not limited to, personal and home care applications, agrochemicals, bioremediation, microbial enhanced oil recovery (MEOR), textiles and biomedical applications. The current leading applications for these compounds is in home and personal care products with many companies utilizing sophorolipids in cleaning and toiletry products and both rhamnolipids and mannosylerythritol lipids in cosmetics and toiletries (Montoneri *et al*., 2009; Onaizi *et al*., 2009; Perfumo *et al*., 2010; Banat *et al*., 2010; Brown, 2010; Vecino *et al*., 2017; Geetha *et al*., 2018).

Due to their low toxicity, good biodegradability and an increase in consumer awareness regarding sustainability and environmental protection, biosurfactants and bioemulsifiers are progressively viewed as alternatives to synthetically produced surfactants (Banat *et al*., 2010). This progressive view of the commercial application of these compounds is seen in the capital value attached to their usage. In 2016, the reported global bio‐based surfactant market was valued at USD 3.99 billion and 460 kilotons with a projected rise by 2022 to USD 5.52 billion and 560 kilotons (Markets and Markets, 2017). A large diversity of microbial species possesses the ability to synthesize surface‐active compounds; however, some of these species are also opportunistic human pathogens. A prime example is the synthesis of rhamnolipids by the Gram‐negative, opportunistic bacterium *Pseudomonas aeruginosa* (Jensen *et al*., 2007). Where this is the case the exploitation of these surface‐active compounds for commercial application becomes complicated due to regulations relating to the cultivation of pathogenic species and also the potential for products obtained from these cultures to be contaminated with harmful virulence factors (Campos *et al*., 2013). Therefore, a large amount of current research is focused on either the identification of microbes producing surface‐active compounds that have non‐pathogenic taxonomic affiliations or engineering surface‐active compound biosynthesis pathways in to non‐pathogenic host strains (Müller and Hausmann, 2011; Müller *et al*., 2011; Funston *et al*., 2016; Twigg *et al*., 2018; Tripathi *et al*., 2019).

In addition to the identification of new biosurfactant‐synthesizing microorganisms, research is also focused on the production of these compounds using renewable and/or waste‐stream‐derived feed‐stocks (Tan and Li, 2018; Wang *et al*., 2019). The outcome of these research avenues is an increasing number of papers reporting biosurfactant production by a wide variety of both prokaryotic and eukaryotic microorganisms. For example, in the marine environment alone there are reports of surface‐active compound production by over 50 different isolates (Tripathi *et al*., 2018). A by‐product of this increased interest in novel surface‐active compound synthesis is the large number of manuscripts that have been published or submitted for peer review that claim either production of a known compound by a ‘new’ microorganism or discovery of previously never described biosurfactants; however, too often these studies lack stringent experimental evidence to convincingly support their claims (Priji *et al*., 2017). This is also true of many studies that claim strain exhibiting enhanced surface‐active compound synthesis or the utilization of novel substrates or process engineering strategies to produce these compounds (Wan Nawawi *et al*., 2010; Nordin *et al*., 2013).

The lack of academic stringency in the literature reporting microbial surface‐active compound production has been crippling the microbial biosurfactants field for a number of years, and was previously highlighted by three publications, which discuss glycolipid biosurfactant production in novel bacterial and yeast strains (Claus and Van Bogaert, 2017; Irorere *et al*., 2017; Roelants *et al*., 2019). This issue was also recently presented by the lead author of this paper at an inaugural international scientific meeting specifically focussing on microbial produced surface‐active compounds; Biosurfactants 2019 held in Stuttgart, Germany. As a result of these publications and the discussion stemming from the conference presentation, a group of global leading researchers in the field of microbial biosurfactants felt that the time was ripe for the publication of definitive protocols appropriate for the reliable publication of claims relating to microbial surface‐active compound production.

In this review, we will critically evaluate the methodologies relating to the identification of surface‐active compound production by microorganisms and the identification of the organisms themselves. While this review primarily focuses on the low molecular weight biosurfactants such as glycolipids and lipopeptides, the techniques discussed here are appropriate for research into high molecular weight bioemulsifier type surface‐active compounds and even further to other types of novel microbially produced biochemicals. We will also highlight some of the issues that require addressing in the scaling‐up of surface‐active compound production and look at techniques that can accurately and reliably establish the functional properties of these compounds. Our overall targets, which we propose to be integrated by the scientific community working on microbiologically produced surface‐active compounds, are as follows: (i) to provide guidelines to the experimental evidence required prior to publication to conclusively establish an organism is producing a surface‐active compound; (ii) suggest a pipeline for the development of a process for surface‐active compound production for commercial application.

## Critical review of experimental phenotypic methodologies for the reporting of surface‐active compound detection

A large number of different experimental techniques are utilized in the studies reporting microbial surface‐active compound production; these range from assaying microbial phenotypic traits associated with surface‐active compound production to chemical analysis methodologies such as high‐performance liquid chromatography (HPLC) coupled to mass spectrometry (MS) (Walter *et al*., 2010). The simplest of these techniques to perform are assays for phenotypic traits indicative of surface‐active compound production. These assays either directly or indirectly detect the presence of surface‐active compounds using either culture broth or supernatant samples obtained throughout the growth cycle of the microbial culture being tested or at a culture end point. Many of these assays measure the effects that surface‐active compounds have on interfacial or surface tension and are generally used for detecting low molecular weight biosurfactant compounds. A tensiometer, for instance one based on the du Noüy ring method, can be applied to a broth or supernatant sample to directly measure surface tension (Du Noüy, 1925). Pendent drop shape, axisymmetric drop shape and drop collapsing assays record the differences in structure of a small volume of sample placed onto a hydrophobic surface due to the presence of biosurfactant compounds (van der Vegt *et al*., 1991; Tadros, 2005; Jain *et al*., 2016). High‐throughput microplate assays have also been developed where a visual distortion effect to a grid pattern placed below the plate, resulting from surface tension reduction due to biosurfactant production, is observed (Chen *et al*., 2007).

**Fig. 1:**
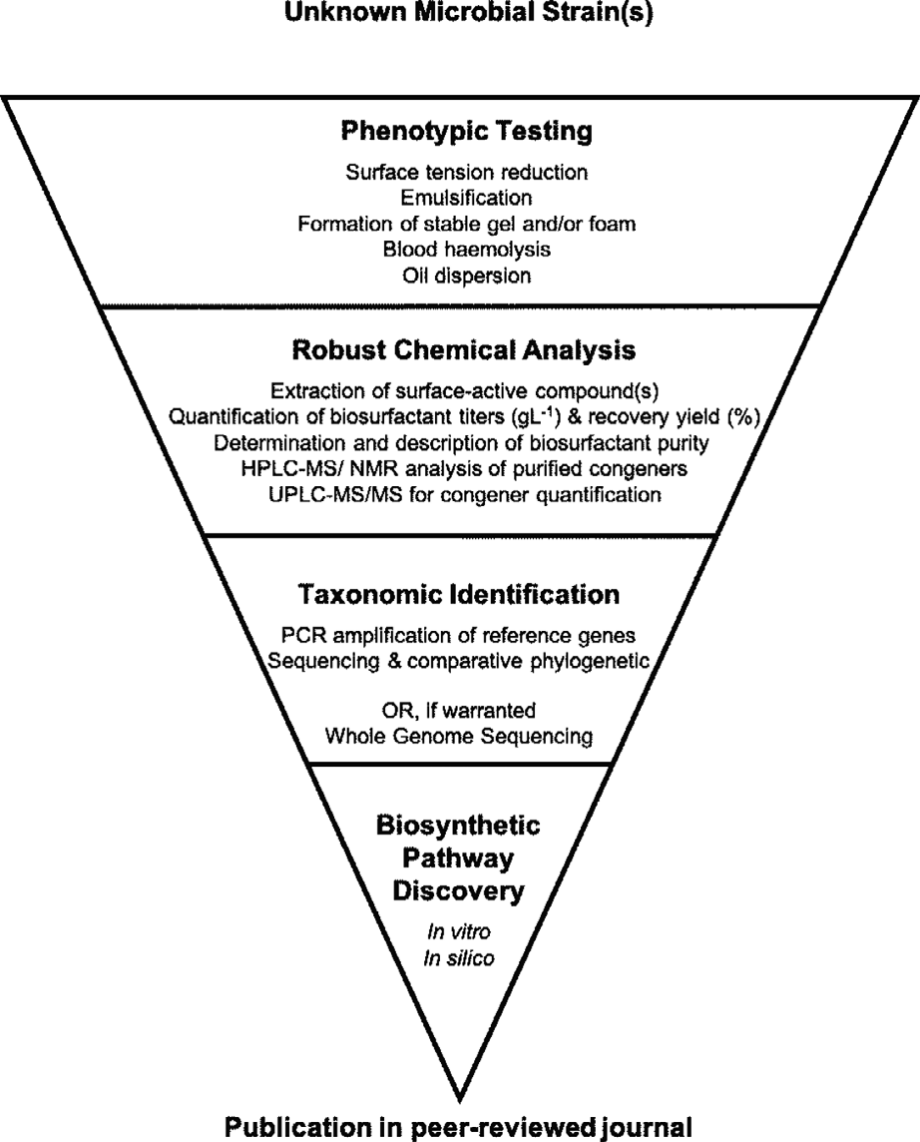
Suggested pipeline for investigating surface‐active compound production by novel microbial species. Published studies should include the phenotypic characterization of traits indicative of surface‐active compound production; robust chemical analysis of purified surface‐active compounds using the techniques listed; accurate taxonomic identification on the strain(s) of using molecular biology methodologies; and an attempt to identify a biosynthetic pathway involved with the surface‐active compound production.

In addition to observing the effect of surface tension changes due to the presence of a biosurfactant in a sample obtained from a microbial culture, other phenotypes associated with surface‐active compounds can be examined. Indirect methods such as measuring the sample’s ability to form emulsions of oil and water can be used to investigate the presence of high molecular weight bioemulsifier compounds, as can the sample’s ability to form stable gels and/or foams (Cooper and Goldenberg, 1987; Lonchamp *et al*., 2019). Another potentially high‐throughput technique is the oil vaporization assay. This versatile method involves spraying a fine mist of oil on the surface of the plates where colonies have been growing, the presence of surface‐active molecules are then detected as a halo of changed light diffraction around a colony (Burch *et al*., 2010). Recently, this methodology has been further improved by including a lipophilic dye in the oil for better contrast (Martinez *et al*., 2020). Surface‐active compounds also have the ability to lyse erythrocytes. This phenotype can be measured by haemolysis assays either on plates, microplates or in micro‐tubes (Mulligan et al., 1977; Manaargadoo‐Catin *et al*., 2016). The advantage of these phenotypic assays is that they are relatively simple to perform; can be adapted to be high throughput to analyse multiple samples; are inexpensive; and, for the most part, do not require specific equipment to perform. However, these phenotypic assays should under no circumstances be solely utilized to confirm surface‐active compound production in a sample. They provide little or no quantification of the concentration of surface‐active compounds present in a sample and absolutely no structural elucidation of any surface‐active compound present in the sample. They can also be very sensitive to interference. For instance, *P. aeruginosa* produces another haemolysin besides rhamnolipids, the secreted enzyme phospholipase C, that will also react will erythrocytes (Lui, 1957; Sierra, 1960; Berka *et al*., 1981). A final problem with the sole utilization of phenotypic assays is that the behaviour of surface‐active compounds differs under specific pH conditions. Therefore, methods based on stabilization of interfaces may render either false‐negative or false‐positive results as an artefact of sample pH. The issue of sample pH interference could be overcome by a relatively new assay format discussed later in this review that produces a colorimetric response to the phenotypic property of surface tension reduction (Kubicki *et al*., 2020). We therefore suggest that these techniques be only utilized at the point of preliminary screening in the pipeline for a study investigating large culture collections of microorganisms for potential surface‐active compound production, or for functional studies with well‐defined microbial strains (Fig. 1). Phenotypic data must always be supported with chemical analysis results before surface‐active compound production can be demonstrated. It should also be always considered that some components of the growth media used for biosurfactant production and the physical condition of the samples may have a significant effect on the surface tension of the medium. Therefore, appropriate experimental controls should be utilized, and statistical analysis carried out to ascertain that the observed effect is due to microbial activity.

Next to the phenotypic methods, the cetyltrimethylammonium bromide (CTAB) assay is popular as well. This agar plate‐based method is utilized in the detection and semi‐quantification of extracellular glycolipid biosurfactants (Siegmund and Wagner, 1991). The basis for the method is the detection of a dark blue halo around a bacterial colony producing and releasing a biosurfactant due to the formation of an insoluble ion pair of CTAB with methylene blue (Siegmund and Wagner, 1991). A major limitation to this assay is that the CTAB reagent, also known as cetrimide, is a broad‐spectrum antimicrobial so therefore can inhibit bacterial growth leading to false‐negative results. Additionally, the assay is non‐specific hence it cannot be accurately used to prove the production of a specific biosurfactant compound. Moreover, even if used as an initial screening method, one must keep in mind that only anionic biosurfactants will be able to form an ion pair.

One of the biggest factors influencing the physico‐chemical properties of this mixture is the presence of lactonic (neutral) and acidic (anionic under alkaline conditions) sophorolipids (Van Bogaert *et al*., 2011). Hence, the results will be biased when a microorganism produces mainly neutral lactonic sophorolipids. Obviously, it becomes even more problematic when strains producing neutral biosurfactants like mannosylerythritol lipids (MELSs), cellobiose lipids and polyol lipids are screened with this method: these producers and their molecules will not be discovered. The drawbacks of the CTAB method are such that we do not recommend its use at all and that researchers should utilize other phenotypic assays such as the aforementioned oil vaporization assay as a replacement technique for initial screening.

## Critical review of experimental colorimetric methodologies for the reporting of surface‐active compound detection

Colorimetric assays measure an absorbance value resulting from a reaction catalysed by the presence of surface‐active compounds in a sample. Two prime examples of such assays used in studying microbially produced glycolipids are the orcinol assay and the anthrone reagent assay for the respective detection and quantification of rhamnolipids and sophorolipids (Hodge and Hofreiter, 1962; Gunther *et al*., 2005; Priji *et al*., 2017). The orcinol assay is routinely utilized in a wide variety of publications to demonstrate and quantify bacterial production of rhamnolipids (Laabei *et al*., 2014). The assay involves adding a solution of orcinol in sulphuric acid to the sample being tested followed by heating; rhamnose sugars present in rhamnolipids will be dehydrated, react with the orcinol to form a green pigmented compound. This compound is measured by recording absorbance at 421 nm (Chandrasekran and Bemiller, 1980). Absorbance readings are then compared with a standard curve generated using samples of known rhamnolipid concentrations or simply rhamnose. The assertion that this assay is sufficient to either prove, or accurately quantify, rhamnolipid production by a bacterial strain is fundamentally flawed. The reason for this view is the significant number of carbohydrate moieties that produce the same reaction with the orcinol reagent as rhamnose. Therefore, unless the initial sample is pure the presence of strong sulphuric acid in the reagent will ensure that any interfering sugars will also give rise to the formation of the green pigment. Potential cross‐reactive sugars include deoxyribose and ribose, both significant components found in nucleic acids and also lipopolysaccharide components present in cellular membranes (De Mey *et al*., 2006; Laabei *et al*., 2014; Pihlasalo *et al*., 2016).

It is worth noting that orcinol assays can become more informative, by including FeCl_2_, then referred to as Bial’s test, to distinguish hexoses from pentoses (Fernell and King, 1953). Therefore, orcinol assays should never be performed on culture supernatant samples but only on samples that have undergone prior extraction with solvent and followed by free sugar/ carbohydrate determination prior to further measurements, for instance as described by Koch *et al*. (1991) and Déziel *et al*. (1996). This way, any aldopentose detected must have been attached to a lipid moiety (glycolipid), since the acidic boiling steps serves to hydrolyse the rhamnolipid and release the sugar part (Koch *et al*., 1991; Déziel et al., 1996). The other major problem with the method is that rhamnolipids are never produced as a single congener but as variable mixtures, usually of mono and dirhamnolipids, therefore even accurate determination of rhamnose concentration is reliant on an estimation step to convert it to a rhamnolipid concentration (Perfumo *et al*., 2013; Pihlasalo *et al*., 2016). Any quantification should always be reported in RE (rhamnose equivalents), as the calibration curve is more reliably and easily prepared with rhamnose (known purity), unless a highly pure rhamnolipid control is available. Based on the inherent flaws with this technique, it is our recommendation that this method should be avoided in any study looking to either prove production in a species not previously reported to produce rhamnolipids. The techniques should also be avoided for rhamnolipid quantification without both sufficient purification and accurate comparative standards.

In a similar way, anthrone reagent is used to quantify sophorolipids in culture liquid or after partial purification. The reagent also contains a strong acid (e.g. sulphuric acid), converting carbohydrates to furaldehydes, which condense with anthrone (9,10‐dihydro‐9‐ozoanthracene) to produce coloured compounds. Absorbance is recorded at 625 nm. Note that likewise as the orcinol method described above the reagent reacts with all carbohydrates including pentoses and sugar alcohols like glycerol and that colour generation and intensity differ regarding the specific sugar involved. Great care is also required to ensure that glassware used in the assay is free from contamination such as paper towel fibres, which are readily hydrolysed by the acid to give a positive result. Hence, it is recommended to only use this assay provided only one type of carbohydrate is present in solution and with a suitable standard (Wang and Cui, 2005). It is hard to meet those prerequisites if using the anthrone method on crude culture broth: other sugars, either derived from the supplemented carbon source or from cell wall components, are also present and not every laboratory uses sophorolipid as a standard for the calibration curve. Hence, practices where sophorolipid concentration is determined by applying the anthrone method to measure total carbohydrate, followed by subtraction of glucose concentration obtained by another method, are questionable (Zhang *et al*., 2018). Nevertheless, both orcinol and anthrone reagent can be used as detection reagent for glycolipids upon thin‐layer chromatography (TLC) analysis, as discussed below.

Although the above descriptions of both the orcinol and anthrone assays have focused on rhamnolipids and sophorolipids; their usage for the detection or quantification of new, as yet undiscovered glycolipid type biosurfactants would present the same inherent drawbacks. Interestingly, Kubicki *et al*. (2020) have recently described a novel colorimetric technique that possesses the potential to both identify and quantify biosurfactant in culture supernatant samples. Unlike both the orcinol and anthrone assays, this assay exploits the surface activity of the biosurfactant and not the chemical nature of the sugar moiety. This is achieved by utilizing Victoria Pure Blue BO dye, which is solubilized due to the actions of the biosurfactant in the sample. Additionally, this assay has been adapted onto a microtitre plate allowing high‐throughput screening of samples. The exploitation of the phenotypic effect of surface tension reduction to produce a colorimetric response would allow this assay to detect multiple different types of biosurfactants (Kubicki *et al*., 2020). However, it should be noted that quantification of biosurfactants using this technique would require the generation of precise standards that had been pre‐validated utilizing a secondary technique. Straightforward spectroscopic methods do exist in the literature to determine the surface activity of molecules based on their micellization and solubilization properties (Kalyanasundaram and Thomas, 1977). These have been applied to both classical surfactants and biosurfactants (Hait *et al*., 2003; Basu Ray *et al*., 2006; Andersen and Otzen, 2014). However, its adaptation to a microtitre plate format is novel and further validation of the assay must be carried out by other independent researchers before being widely utilized by the microbial biosurfactants community.

## Critical review of analytical chemistry methodologies for the reporting of surface‐active compound detection

Utilization of analytical chemistry techniques to prove and characterize surface‐active compound production is significantly more conclusive than both the phenotypic and colorimetric tests discussed above. These techniques include thin‐layer chromatography (TLC), Fourier transform infrared spectroscopy (FTIR), high‐performance liquid chromatography‐mass spectrometry (HPLC‐MS), tandem mass spectrometry (MS/MS) and nuclear magnetic resonance spectroscopy (NMR). All these techniques require the (partial) purification of surface‐active compounds from cell‐free supernatant samples. Methodologies for the purification of surface‐active compounds are often defined by the type of compound predicted to be produced by the microorganism being investigated and are discussed elsewhere (Heyd *et al*., 2008). It is however important to note that to obtain a meaningful characterization of surface‐active compounds one must carefully consider sample purity, as different techniques require differing levels of sample purity. Sample purity is also an important consideration when carrying out functional testing of surface‐active compounds and in designing scale‐up protocols for commercial application. Both these points are discussed later in this review.

By loading supernatant samples or solvent extracts obtained from microbial cultures to silica gel TLC plates and applying a solvent mobile phase composed, for instance, of chloroform, methanol and acetic acid amphiphilic molecules can be separated. In the case of glycolipids (rhamnolipids, sophorolipids, cellobiose lipids and MELs), these are typically detected on the TLC plate colorimetrically using for example the above‐mentioned reagents orcinol or anthrone (Asmer *et al*., 1988; de Koster *et al*., 1994; Hewald *et al*., 2006; Das *et al*., 2014). This method can be utilized to distinguish relatively large differences in glycolipid structure (i.e. between mono‐rhamnolipid and di‐rhamnolipid congeners and lactonic and acidic sophorolipids), as the samples can be compared with standards composed solely of each type of rhamnolipid and/or a mixture of the two (Christova *et al*., 2004). However, TLC can provide little further detail on congener composition and is poor in quantifying the amounts/ titres of glycolipids present in a sample (Van Renterghem *et al*., 2019).

Fourier transform infrared spectroscopy (FTIR) has been proposed as a rapid technique allowing the identification of surface‐active compounds and their quantification (Leitermann *et al*., 2008). This technique has been utilized in a number of studies reporting glycolipid production by various bacterial strains (Nalini and Parthasarathi, 2014). However, there are significant drawbacks to solely utilizing FTIR to conclusively identify the type of surface‐active compound being produced by a microorganism. The principle of FTIR is that the various chemical bonds present in the surface‐active compound produce a specific spectrum that can be detected when analysing a sample by comparison to a known standard composed of the specific compound or to standards that have analogous chemical groups (Leitermann *et al*., 2008). However, as these chemical bonds are not unique to the surface‐active compound and can be present in a large number of other extracellular compounds produced by the microorganisms the sample is required to be of a high level of purity to allow specific detection.

Many studies utilize FTIR on either crude or poorly purified cell‐free supernatant extracts that contain either unutilized media components or other unrelated microbial extracellular products, therefore potentially leading to false positive detection. A second drawback to this technique is its inability to fully characterize the molecular structure of various surface‐active compounds being produced by a single microbial species. Rhamnolipid‐producing bacteria such as *Pseudomonas* and *Burkholderia* species and sophorolipid producing yeasts such has *Starmerella bombicola* do not produce one single type of these respective biosurfactants but produce a range of different congeners (Tulloch *et al*., 1968; Asmer *et al*., 1988; Déziel *et al*., 1999; Haba, Abalos, *et al*., 2003; Gunther *et al*., 2005; Elshafie *et al*., 2015). ATR‐FTIR is limited in its ability to differentiate between these similar structure congeners and therefore does not provide a comprehensive characterization of the biosurfactants being produced by the strain(s) of interest.

Combined with high‐resolution mass spectrometry and tandem mass spectrometry (discussed below), nuclear magnetic resonance spectroscopy (NMR) is the gold standard method to determine the chemical structure of new or unknown biosurfactant molecules, NMR having been used in the field since the 1960s (Tulloch *et al*., 1968; Asmer *et al*., 1988). This combination of techniques continues to provide a highly robust analysis of surface‐active compound production, and were used by studies investigating marine bacteria not previously known to produce biosurfactants, and to characterize biosurfactant compounds produced by engineered microbial strains (Twigg *et al*., 2018; Van Renterghem *et al*., 2018; Roelants, *et al*., 2018; Tripathi *et al*., 2019). Similar to FTIR, NMR also detects the signatures of chemical species relating to the presence of surface‐active compounds in a sample though is generally considered a more robust method of analysis and is the only technique to accurately attribute the nature and positioning of the sugar moiety (Agrawal, 1992). However, as with FTIR one must ensure that the sample being analysed is of a high level of purity or otherwise unused media components and/or unrelated extracellular compounds will be detected and interfere with the results obtained. Additionally, the equipment required to carry out NMR analysis of samples comes at a relatively high financial cost and requires experienced personnel to analyse the generated spectra, this can of course be overcome by collaboration with researchers in the field who have access to core facilities possessing the necessary apparatus and expertise.

In a collaborative study recently published, NMR was shown to be a highly valuable tool in the characterization of a 15‐membered macrodilactone‐containing glycolipid being produced by *Pantoea anantis* (Gauthier *et al*., 2019). Interestingly this novel biosurfactant was being synthesized by the action of RhlAB orthologues, enzymes usually involved in rhamnolipid biosynthesis (Ochsner *et al*., 1994; Smith *et al*., 2016; Gauthier *et al*., 2019). NMR therefore remains the most powerful technique which unambiguously resolves the molecular structure when combined with purification methods such as preparative chromatographic methods or fraction collection with HPLC to obtain pure compounds (Van Bogaert *et al*., 2016). Sample purification in order to perform accurate NMR analysis could be seen as major drawback of this technique. The level of purification needed often requires some pre‐requisite knowledge of the general structure of compound(s) being produced (i.e. rhamnolipid, sophorolipid, lipopeptide); as such, NMR analysis may not be suitable for high throughput analysis of multiple strains of interest. Additionally, high‐resolution NMR requires the use of a good solvent, which may be tedious to find for high molecular weight surface‐active compounds.

As just mentioned above in order to fully characterize the different congeners of surface‐active compounds being produced by a microbial isolate of interest, one must utilize a technique that has the ability to separate and individually analyse each congener. The separation of the different congeners is typically achieved using high‐performance liquid chromatography (HPLC). This separation technique is then ideally combined with the use of mass spectrometry (MS) for detection and analysis or each congener (Haba, Abalos, *et al*., 2003; Smyth *et al*., 2014). Matrix‐assisted laser desorption ionization‐time of flight (MALDI‐TOF) can also be used to characterize the structure of biosurfactants (Price *et al*., 2009). More recently, the use of MALDI‐TOF has emerged as an approach for the discovery of new microorganisms producing biosurfactants, which can then include structural characterization in a screening strategy (Kurtzman *et al*., 2010; Sato *et al*., 2019). When analysing low molecular weight biosurfactants such as rhamnolipids and sophorolipids, HPLC‐MS is considered to be the most precise and versatile methodology to use. There are a number of studies that have comprehensively analysed a wide variety of different glycolipid congeners using HPLC‐MS, and in this way, results from the analysis of samples obtained from cultures of microbes of interest can be readily compared and assigned structures (Déziel *et al*., 1999; Haba, Abalos, *et al*., 2003; Rudden *et al*., 2015).

Further structural elucidation can be carried out utilizing tandem mass spectrometry (MS/MS) where a single congener can be fragmented and the resultant daughter ions analysed (Dubeau *et al*., 2009; Tripathi *et al*., 2019). This technique provides further stringency to the characterization of surface‐active compounds being produced by novel microbial strains and has been recently utilized to provide confirmatory evidence of rhamnolipid production by a species of *Marinobacter*, further expanding the paradigm of rhamnolipid production into a new genus of commonly isolated marine bacteria (Tripathi *et al*., 2019). In a similar way, Price *et al*. (2009) performed a detailed structural characterization of novel sophorolipids from several new members of the *Starmerella* clade. They used MALDI‐TOF‐MS to determine the different glycolipid profiles among the different species and characterized some novel compounds by combining this with carbohydrate and lipid analysis utilizing gas chromatography (GC)‐MS and NMR spectroscopy (Price *et al*., 2009).

Due to the unreliability of quantification methods used in many studies and lack of use of reliable standards, many claims for titres and yields of biosurfactants are often wildly exaggerated (Li *et al*., 2019, 1980). As this information is critically important, for instance for comparison purposes and if commercial production is to be achieved, this is a matter of extreme concern (Roelants *et al*., 2019). Therefore, in addition to providing a full structural characterization of surface‐active compounds being produced by a strain of interest, HPLC‐MS techniques have been developed to provide an assessment of the abundance of each congener being synthesized, providing this important quantitative determination of compound production. Early on, Déziel *et al*. (2000) reported the use of a collision‐induced dissociation tandem MS method combined with direct injection of culture supernatants to precisely detect and quantify rhamnolipids. Since then several variations and refinements have been reported (Déziel *et al*., 2000). For instance, Rudden *et al*. (2015) developed an ultra‐performance liquid chromatography tandem mass spectrometry (UPLC‐MS/MS) technique, which provided an accurate quantitative determination. This methodology was validated by the analysis of both a commercially available rhamnolipid preparation and cell‐free supernatant extracts of *P. aeruginosa* ST5 cultures (Rudden *et al*., 2015). More recently, atmospheric pressure chemical ionization mass detection (APCI‐MS) has also gained interest as a simpler and more affordable option when the chemical nature of the molecules of interest is well characterized (Ratsep and Shah, 2009). It should be emphasized that consistent quantification is only achieved when using a reliable internal standard (Abdel‐Mawgoud *et al*., 2014). In the case of sophorolipids, pure standards can be used to quantify total sophorolipid amounts and major congener abundance (Roelants *et al*., 2016). These standards can either be obtained commercially (e.g. Carbosynth, UK) or be created by the researcher, for example by chemical synthesis or purification from culture supernatant (note that proper analysis and validation remains a requirement).

Considering the above we therefore consider HPLC/UPLC‐MS techniques to be the gold standard in the analysis (qualitative and quantitative) of surface‐active compound production; the structural characterization of such components and the quantitative determination of yield (Fig. 1). When available, and only when combined with suitable techniques to obtain purified congeners further stringent structural elucidation can be achieved with NMR techniques.

## Critical review of experimental methodologies for the reporting of strain identification

Another prevailing issue has been reports of (apparently) new microbial species producing (often already known) biosurfactants. Therefore, in parallel to a requirement for stringent experimental evidence specifically characterizing surface‐active compound production by any microorganisms, there is an equally important need for accurate determination of the identity of that organism. It is only with this information on taxonomic identity that a study can be judged as being completely new to science. As with assignment of the type of surface‐active compounds being produced by an isolate of interest, there are many methodologies available to achieve taxonomic classification. These methods broadly fall into two general categories: culture‐driven and molecular biology‐driven methodologies.

Culture‐driven methodologies consist of the observation of colony morphology of bacterial or yeast cultures; the observation of cellular morphology using microscopy following various staining techniques (i.e. in the case of bacteria, Gram staining); and a combination of biochemical assays such as Analytical Profile Index (API) tests. Studies reporting surface‐active compound production in novel microbial strains have been published where culture‐driven approached have been solely utilized for the identification of the strain being described (Tuleva *et al*., 2002; Chen *et al*., 2006; Toribio *et al*., 2010; Bendaha *et al*., 2012; Nordin *et al*., 2013). The use of these techniques to identify microbial strains presents a significant problem as in most cases they are not stringent enough to accurately define a microorganism at species level. Utilization of either colony and/or cellular morphology can only provide a rough identification of a microorganism. Combining these observations with API tests improves the degree of taxonomic identification. However, most of these API tests are only validated for usage in clinical microbiology labs for the identification of pathogenic species and therefore are significantly limited with regard to the range of organisms characterized by the tests. This is problematic as the majority of studies reporting surface‐active compound production are investigating strains of microorganisms isolated from various environmental niches. A pertinent example where reliance on culture‐driven strain identification has caused problems was in the publication of novel sophorolipid synthesis by *Wickerhamiella domercqiae* (Chen *et al*., 2006). This study utilized BIOLOG assays combined with observation of colony/ cell morphology to assign species. However, whole‐genome sequencing (WGS) of this yeast in fact identified it as *S. bombicola*, a species already well characterized as being a sophorolipid producer (Li *et al*., 2016). In this case, misidentification on the strain called into question a number of subsequent publications and potential patent applications (Chen *et al*., 2014).

By far, a more accurate methodology of identifying microbial strains is utilizing molecular biology‐driven techniques, and it is these techniques that should be utilized for the taxonomic classification of surface‐active compound producing microorganisms (Fig. 1). Microbial genomes incorporate elements that consecutively possess domains with conserved and variable sequence, and the variable sequence domains are often strain‐dependent and can therefore be utilized to phylogenetically type the organism. Such genetic elements are commonly referred to as reference sequences. In bacteria, the most ubiquitous example is the gene encoding the 16S subunit of ribosomal RNA (16S rRNA) (Langille *et al*., 2013). Other commonly utilized phylogenetic reference genes in bacteria are the DNA gyrase B gene (*gyrB*) and the gene encoding the RpoD RNA polymerase sigma factor (*rpoD*) (Yamamoto and Harayama, 1995; Mulet *et al*., 2009). In eukaryotic microorganisms, the internal transcribed spacer (ITS) region in the 18S subunit of ribosomal RNA is often used (Schoch *et al*., 2012). Alternatively, the D1/D2 domains of the large subunit (LSU) rRNA can be used as well, as demonstrated by Kurtzman *et al*. (2010) to identify several novel sophorolipid producing yeast species (Kurtzman *et al*., 2010). Reference genes are PCR‐amplified using universal primers that bind to the conserved sequence domains. Examples of such universal primers include 9bfm, 27F, 341F, 534R, 1492R and 1512uR for the amplification of the 16S rRNA gene and ITS1–ITS4 for the amplification of the ITS region of 18S rRNA (Watanabe *et al*., 2001; Baker *et al*., 2003; Martin and Rygiewicz, 2005; Mühling *et al*., 2008). Resultant amplicons are then sequenced and compared, via Basic Local Alignment Search Tools (BLAST) to nucleotide sequence databases; these can be general ones or databases dedicated to ribosomal RNA like offered by SILVA (https://www.arb‐silva.de) (Altschul *et al*., 1990; Quast *et al*., 2013). With regard to the identification of bacteria utilizing the 16S rRNA reference gene, specific 16S databases such as RDP (https://rdp.cme.msu.edu) and EzBioCloud (https://help.ezbiocloud.net/ezbiocloud‐16s‐database) are recommended (Cole *et al*., 2011; Yoon *et al*., 2017).

Although sequencing of reference genes provides a significant improvement in the accuracy of microbial identification compared with culture‐based methodologies, reference gene sequencing can only be used to assign an unknown microbial strain to the genus level, for some species of bacteria. However, these sequence data can be compared with similar data from type strains of the different species within the genus via multiple sequence alignment to generate a phylogenetic tree showing the relatedness of the strain of interest to the various species within the genus. This type of phylogenetic analysis can be achieved utilizing the alignment, classification and tree tools housed by SILVA (Pruesse *et al*., 2007; Quast *et al*., 2013). This methodology was utilized by Twigg and co‐workers to show that a rhamnolipid‐producing marine bacterial strain identified as belonging to the genus *Pseudomonas* was not related to *P. aeruginosa* or any of the other already reported rhamnolipid‐producing *Pseudomonas* species (Twigg *et al*., 2018). Thus, a combination of different reference genes can be used to improve the accuracy of phylogenetically typing of a strain of interest, as was shown in the identification of two rhamnolipid‐producing marine bacteria where both the 16S rRNA gene and *gyrB* were used (Twigg *et al*., 2018; Tripathi *et al*., 2019). Moreover, information on the phylogenetic position of a biosurfactant‐producing yeast can provide a hint to the type of compound synthesized. Strains belonging to the *Starmerella* clade most likely produce sophorolipids or related compounds, while yeasts of the *Ustilagomycotina subphylum* have a higher likelihood to synthesize mannosylerythritol lipids and/or cellobiose lipids. If the basidiomycete belongs to the *Sporidiobolales* order, polyol esters could be the compound of interest (Claus and Van Bogaert, 2017). Nevertheless, the exact structure always requires proper confirmation by one of the methods described above.

To further improve the degree of microbial identification above the level of accuracy obtained from the sequencing of phylogenetic reference genes one can employ WGS. The feasibility, with regard to cost and time, of WGS to type a novel strain of interest has greatly improved since the advent of next generation sequencing platforms such as Illumina sequencing, Nanopore sequencing and Single Molecule Real Time (SMART) sequencing (Bentley *et al*., 2008; Eid *et al*., 2009; Jain *et al*., 2016). Although the financial cost of carrying out WGS is greater than that of amplicon sequencing and the resultant data require a greater degree of bioinformatic expertise to process, the degree of taxonomic identification is a lot greater. Additionally, WGS of a novel surface‐active producing strain of interest can provide significantly more information about the strain and may aid in the identification of genetic elements responsible for the biosynthesis of surface‐active compounds. The authors of this present study do not recommend that WGS be used in all future studies to identify surface‐active compound producing microbes, but that WGS techniques should not be discounted.

## Elucidation of surface‐active compound biosynthesis pathways

The final piece of information required to provide stringent proof that a novel microbial strain of interest is producing surface‐active compounds would be evidence of the biosynthetic pathway/ enzymes the organism is utilizing for production (Fig. 1). In the case of rhamnolipid production by bacteria strains, this pathway is relatively straightforward. Mono‐rhamnolipids are produced via the actions of two enzymes RhlA and RhlB which, respectively, catalyse the formation of a fatty acid precursor moiety (3‐(3‐hydroxyalkanoyloxy)alkanoic acid; HAA) and then conjugates HAA to dTDP‐rhamnose (Ochsner *et al*., 1994; Déziel *et al*., 2003). A second rhamnosyltransferase (RhlC) then utilizes the mono‐rhamnolipids as a substrate conjugating a second dTDP‐rhamnose to form di‐rhamnolipid (Rahim *et al*., 2001). These three enzymes are encoded by three genes: *rhlA*, *rhlB* and *rhlC* (Ochsner *et al*., 1994). Orthologues of these three genes are present in the well‐characterized rhamnolipid‐producing strains of *P. aeruginosa* and a few *Burkholderia* species, such as *B. thailandensis*, *B. pseudomallei* and *B. glumae* (Ochsner *et al*., 1994; Dubeau *et al*., 2009; Costa *et al*., 2011; Funston *et al*., 2016). Interestingly in *P. aeruginosa, rhlA* and *rhlB* are found in a single operon alongside an AHL‐mediated quorum sensing system (*rhlI*/*rhlR*) with *rhlC* located separately, while in the *Burkholderia* species all three genes are located on single operons, intriguingly duplicated in *B. thailandensis* and *B. pseudomallei*, without a nearby quorum sensing system (Dubeau *et al*., 2009).

The *Pseudomonas* and *Burkholderia* biosynthetic rhamnolipid pathways share up to 48% sequence similarity at both the genetic and amino acid level (Dubeau *et al*., 2009; Funston *et al*., 2016). As this biosynthesis pathway is only formed from the expression products of three genes, it is not unreasonable to expect presentation of evidence that attempts to identify these genes when publishing a study reporting rhamnolipid production in a novel strain. This can be achieved by using either *in vitro* or *in silico* methodologies. The *in vitro* approach utilizes *rhlA‐C* sequence data from well‐characterized rhamnolipid‐producing strains to generate sequence alignments for the identification of conserved regions that can then be used to design primers. These primers are used to screen chromosomal DNA extracted from isolates of interest via PCR. This *in vitro* methodology was utilized in the study of rhamnolipid‐producing marine bacteria (Twigg *et al*., 2018; Tripathi *et al*., 2019). These conserved regions of either DNA or amino acid sequence can also be utilized to carry out an *in silico* probe of either the sequenced genome of the strain of interest or previously published genomes of closely related strains, again this approach was used by Tripathi *et al*. (2019).

PCR‐based amplification of biosynthetic genes in glycolipid producing yeast is less straightforward. Although the genes display homology, this is insufficient for the design or (degenerated) primers or probes. Nevertheless, more WGS have become available, also for ascomycetes and the more complex basidiomycetes species like the mannosylerythritol lipids, cellobiose lipid and polyol producers. For most of the well‐described compounds, a reference synthetic gene cluster is available, and recently, also the first biosynthetic pathway for the polyol liamocin was described (Jezierska *et al*., 2018; Xue *et al*., 2020). Screening genomes of novel producers with a query based on these known clusters or, in, for example, in the case of liamocins, based on retrieving polyketide secondary metabolites signatures is also an effective approach. This method works very well for retrieving mannosylerythritol lipids genes, as the producers are found in a tight taxonomic group, and the cluster format seems to be quite conserved. Nevertheless, albeit many cellobiose lipids clusters can be retrieved in a similar way, these come with higher conformational variation combined with an occurrence in a wider taxonomic group besides members of the mannosylerythritol lipids producing *Moesziomyces*, also certain *Trichosporon* and *Cryptococcus* strains are reported to produce cellobiose lipids (Pyatt *et al*., 2018). Just like mannosylerythritol lipids, sophorolipids are retrieved in a narrow taxonomic group, the *Starmerella* clade, so also here putative clusters can be retrieved. Nevertheless, exceptions are possible. Upon analysis of the WGS of *Candida apicola*, a reported producer, no sophorolipid biosynthetic gene cluster could be retrieved and the same holds true for the cellobiose lipid producer *Moesziomyces* (former *Pseudozyma) aphidis* (Morita *et al*., 2013; Vega‐Alvarado *et al*., 2015). A multitude of different software tools exist for the retrieval of biosynthetic clusters, examples used in the field of biosurfactant research include PRISM and anti‐SMASH (Xue *et al*., 2020). However, a complete description of these *in silico* techniques would be the premise of an independent review. Further information regarding genome mining techniques and their application in natural product discovery can be found in reviews by Machado *et al*. (2017) and Ziemart *et al*. (2016).

Finally, functional genomics approaches represent an important aspect of any forward‐thinking research on metabolite biosynthesis. Identification or confirmation of genes involved in biosurfactant production can generally be performed by random or targeted mutagenesis, in amenable microorganisms. Hence, the initial discovery of *rhlAB* genes in *P. aeruginosa* was achieved by screening random transposon mutagenesis libraries, using the above‐mentioned CTAB plates, for rhamnolipid‐defective mutants (Ochsner *et al*., 1994). Confirmation that both operons coding for *rhlA*, *rhlB* and *rhlC* genes in *B. thailandensis* are functional and contributing to the total production of the same rhamnolipid congeners was only possible by the inactivation of the respective *rhlA* genes (Dubeau *et al*., 2009). Ideally, this would be accompanied by cloning the biosynthetic genes on an expression vector for complementation of the mutants, or even heterologous production in a new microbial host (Ochsner *et al*., 1995; Cabrera‐Valladares *et al*., 2006; Wittgens *et al*., 2017; Dulcey et al., 2019). Achieving both biosynthetic gene inactivation and controlled production via expression vectors represent the ultimate demonstration that biosynthetic genes have been identified, and serves as a basis for future metabolic engineering strategies.

## Addressing inconsistencies and errors in the use of terminology with in microbial biosurfactant research

A major issue with many reports focussing on microbial biosurfactant production and subsequent process development is the use of incorrect terminology and the lack of reporting of important process parameters and outputs. Concerning terminology an ‘amount (g)’ of product should be defined as the weight gravimetrically determined after purification (possibly still containing other compounds, like proteins, residual hydrophobic molecules, proteins etc. (see above)). A ‘titre (g L^‐1^)’ is defined as the concentration of a product. Logically, the end titre corresponds with the concentration when the fermentation is terminated and can be determined as described above.

In a majority of papers focusing on microbial biosurfactants, the end titre is confused with ‘yield (%)’. Yield is defined as the percentage of product produced from a certain amount of substrate in a fermentation: it is the amount of surface‐active compound produced (g) divided by the total amount of hydrophilic and hydrophobic substrate consumed by the microorganism. Generally, yields between 0.3 and 0.7 g g^‐1^ are obtained for Sophorolipid production although higher efficiency levels are sometimes reported, the reader must always remain critical and carefully consider the validity of the methods that were used to reach the reported conclusions (Van Bogaert *et al*., 2007; Roelants *et al*., 2016; Van Renterghem *et al*., 2018, Roelants, *et al*., 2018; Li *et al*., 2019, 1980). Yield (%) can also refer to the product recovery after purification. In future reports the correct terminology should be used. Finally, another very important parameter the **‘**productivity (g h^‐1^) or volumetric productivity (g L^‐1^ h^‐1^)’, which is one of the main denominators for fermentations and also often an important denominator for production costs/kg of product (Van Renterghem *et al*., 2018). Typically, this value is not reported, although it can be easily deduced from the end titre and the duration of the fermentation process. A microbial biosurfactant concentration at the end of a fermentation alone is of little value, as very high titres can be obtained over a very long‐time span, thus seriously decreasing productivity and increasing cost of goods (COG). Similarly, a very high final concentration can be obtained, but with a very low carbon conversion efficiency (low yield). In conclusion, end titres, yields and productivities should ideally be clearly defined and the correct terminology applied. A last terminology‐related issue is the difference between ‘purity’ and ‘uniformity’ which is also sometimes confused. As mentioned above most microbial biosurfactants are produced as a complex mixture of congeners. This results in a non‐uniform biosurfactant product, while contaminants like proteins, sugars, oils, fatty acids, cells and water are often associated with purified biosurfactants, thus lowering their final purity.

## Process development towards the scale‐up and commercial application of microbial surface‐active compounds

Process development (optimization of fermentation and purification methodologies) and scale‐up are the next stages that should typically follow once the aspects described above have been carefully considered. It is essential before one can start investigating the optimization of process conditions to improve the production and purification of the microbial biosurfactants of interest. This process development requires appropriate and validated analytical methods, preferably utilizing highly pure standards of the product of interest. These standards are used for accurate qualitative and quantitative analysis. Qualitative analysis most importantly refers to the relative abundance of the varying congeners present in the biosurfactant samples. As mentioned above, microbial biosurfactants are typically a mixture of similar congeners, for example mono‐ and di‐RL or lactonic and acidic sophorolipids (Abdel‐Mawgoud *et al*., 2014; Roelants *et al*., 2016). Often due to the amount of different congeners being produced, it is highly impractical to generate and use individual standards of all the separate biosurfactant congeners for quantification.

During process development, we recommend to generate highly pure standards of the biosurfactant mixture and then use these ‘standards’ (consisting of a mixture of congeners) to analyse broth samples containing the same mixture of biosurfactant congeners in the same ratio’s (Roelants *et al*., 2016; Lodens *et al*., 2020). Important to note is that one should carefully consider the ratio’s of the compounds/peaks in the samples versus the standards. If these ratio’s remain the same this method is valid. Because the latter is not always the case, it is also recommended – if possible – to generate highly pure standards of the most abundant congeners available in the mixture, so these can be quantified (absolutely) separately, for example non‐acetylated C18:1 acidic sophorolipids, di‐acetylated C18:1 lactonic sophorolipids, non‐acetylated C18:1 acidic glucolipids (Roelants *et al*., 2016; Lodens *et al*., 2020). This will thus be a case to case issue, but the authors of this work active on these aspects, typically make sure both options are at hand. These highly pure biosurfactant standards can be generated either from the organism of interest, from an already well‐described microbial organism (i.e. *P. aeruginosa* in the case of rhamnolipids and *S. bombicola* in the case of sophorolipids) or purchased from a third party. Synthetic rhamnolipids are also becoming available and could represent an interesting avenue for standards (Compton *et al*., 2020). The generated standards are used to follow up the concentration of the microbial biosurfactant of interest during fermentation and purification experiments, but also to quantify the biosurfactant in new purified biosurfactant samples and hence determine the overall purity of the new products (Roelants *et al*., 2016). It should be mentioned that the generation of such highly pure standards requires some time and expertise on purification – and analytical aspects, as discussed previously. Therefore, most authors unfortunately do not invest in this and use rather crude and mostly inaccurate methods for quantification as described below.

The same situation applies to rhamnolipids – it is impractical to define response factors to be used in LC/MS analyses for each individual congener in the mixtures produced during fermentations, and therefore, only the most abundant ones are generally considered (Abdel‐Mawgoud *et al*., 2014). We advise at the same time to have validated analytical methods available for the substrates used for microbial biosurfactant production. Typically, hydrophobic (such as fatty acids, plant oils) and/or hydrophilic (such as carbohydrates, polyols) substrates are used, for which highly pure standards are available. The monitoring of substrate concentration in the culture medium throughout the fermentation is often a key step in the optimization of microbial biosurfactant production (Van Bogaert *et al*., 2007; Funston *et al*., 2017; Tripathi *et al*., 2019).

A highly important consideration when optimizing process development and designing scale‐up strategies for the biotechnological application of a surface‐active compound is the calculation of production yields and the determination of product purity. In the field of microbial biosurfactant research a lot of researchers revert to the use of ‘extraction’ methods combined with gravimetrical determination of ‘purified’ samples of microbially produced surface‐active compounds. Such methods are widely applied for both glycolipid type biosurfactants and high molecular weight bioemulsifier type compounds (Kourmentza *et al*., 2019; Naughton *et al*., 2019; Roelants *et al*., 2019). When performing biosurfactant purification via liquid‐phase extraction followed by evaporation of the solvent or by precipitation of the compounds from broth or supernatant the end point is often an oily, honey‐like product (Roelants *et al*., 2016; Çakmak *et al*., 2017). These oily products typically also still contain up to 60 % water, which is mostly not determined and/or reported and thus results in an overestimation of the reported production.

The use of laboratory equipment, commonly utilized to determine cell dry weight of broth samples in microbial laboratories, can address this issue. The substrates used in the fermentation, especially the hydrophobic substrates such vegetable oils and derived fatty acids, are also typically contaminants in final reported ‘purified’ microbial biosurfactant samples. Huge overestimations of the reported produced biosurfactants can occur if the presence of these contaminants is not taken into account (Roelants *et al*., 2019). To give an example, precipitation of sophorolipids from fermentation broth (e.g. by heating up the broth to 60°C and subsequent washing), results in an oily sophorolipid product. In many reports this is considered as 100 % sophorolipids while in fact this crude sophorolipid product can typically contain up to 20 % fatty acids and oil, 60 % water (as mentioned above) and 10 % of other impurities such as medium components (e.g. salts and carbohydrates, proteins, DNA) (Roelants *et al*., 2019). The actual sophorolipid content of such sample would thus only amount to 20 % of the total sample resulting in a huge overestimation of produced mounts. On the other hand, this final measurement will always be an underestimation of what was ‘actually’ produced during growth as product will always be lost throughout the purification process.

The various types of contaminants to consider depend on the medium components and substrates used during the fermentation and the type of microorganism being utilized to produce the desired surface‐active compound. Contaminants that can be present in a so‐called ‘purified sample’ include proteins, which can be determined using standardized methods such as BCA or the determination of total nitrogen using Kjeldahl methodologies; carbohydrates, for which analytical methods are widely available and described; fatty acids and oils, for which typically GC methodologies are applied; salts, for which determination of the ash content is a good measure; endotoxins, for which commercial kits are available (Bremner, 1960; Walker, 1994; Dodds *et al*., 2005; Ohemeng‐Ntiamoah and Datta, 2018). Finally, the determination of contaminating DNA becomes an important factor further along the innovation chain, aiming for commercialization and is less important at initial stages which can, for example, be determined using (quantitative) PCR methodologies.

When product samples are not only generated for quantification reasons as mentioned above, but also towards further evaluation, for example, critical micelle concentration (CMC), assembly, emulsifying properties, it is even more important to consider purity and only work with highly pure products, which purity should also be reported. The presence of contaminants such as fatty acids, fatty acid methyl esters or oils used as substrates in fermentation media, in the final product can dramatically affect the physico‐chemical and biological properties of the generated biosurfactant product. When evaluating biosurfactant samples of low purity, the resulting properties will not be linked to the biosurfactant alone, but to the biosurfactant in a mixture with a range of contaminants. Moreover, the ratio of the biosurfactant congeners is also extremely important here and should also be reported. The absence thereof has resulted in highly confusing reports of, for example, ‘sophorolipids’, which have foaming properties in one publication, while they do not foam at all in another one, which is quite probably due to the fluctuation in the ratio of acidic and lactonic sophorolipids between reports. This heterogeneity in sophorolipid samples was also addressed as a possible explanation to the different aggregation behaviour (ribbons against micelles) in water (Dhasaiyan *et al*., 2017). Again, the use of validated analytical methods, as described and recommended above, allows authors to report on these ratio’s together with sample purities and linked with tested properties. We thus reiterate our recommendation for the use of highly pure standards for analysis of microbial biosurfactants in various samples as described above taking into account the ratios of the biosurfactant congeners and reporting on these aspects. When this is not possible and authors use crude gravimetrical methods, our guidelines are to rigorously analyse the resulting biosurfactant samples for contaminants (qualitative and quantitative) and provide an estimate about product loss during purification.

## Function testing methodologies for assigning the application of microbial surface‐active compounds

Once samples of high‐enough purities are obtained for a surface‐active compound of interest, the physico‐chemical properties of the molecules should be carefully investigated. Rigorous characterization of the solution properties of surface‐active molecules is crucial in view of their applications. To achieve this there are three major questions which one should answer: (i) at what concentration do they aggregate; (iI) what is the morphology of the individual aggregates; (iii) what is the structure of a single and of a collection of aggregates. To address these questions in colloids science terms, one speaks of surface tension and critical micelle concentration, solution self‐assembly and phase behaviour. These aspects have been commonly addressed in a number of key publications over the past seven decades (Griffin, 1946; Davis, 1957; Israelachvili *et al*., 1976; Tanford, 1980; Bergström, 2007). The systematic analysis of surface‐active molecule application has been performed with a number of complementary experimental techniques. In this section, we provide a description of the most common and suitable techniques applied to the study of self‐assembly in solution.

Differently from the previous sections, we assume the use of a homogeneous and pure microbial surfactant sample, dispersed in a solvent (generally water) at known concentration. We also indicate the level of accessibility and experience required to perform these analyses. In general, for a representative insight on the solution aggregation of surface‐active compounds, several aspects should be respected. These include performing the study in the parent solvent; avoiding undesired variations in concentration due to, for example, dilution or drying; avoiding sample degradation; adapting the technique to the dynamics of self‐assembly, being aware of common artefacts and, very importantly, combining several techniques in the analysis. In this regard, we concentrate on the aggregation behaviour in solution and do not consider a large family of analytical techniques commonly employed to probe self‐assembly at liquid‐air or solid‐air interfaces (e.g. Brewster angle microscopy, X‐ray photoelectron spectroscopy, X‐ray and neutron reflectivity, fluorescence recovery after photobleaching microscopy and many others).

**Fig. 2:**
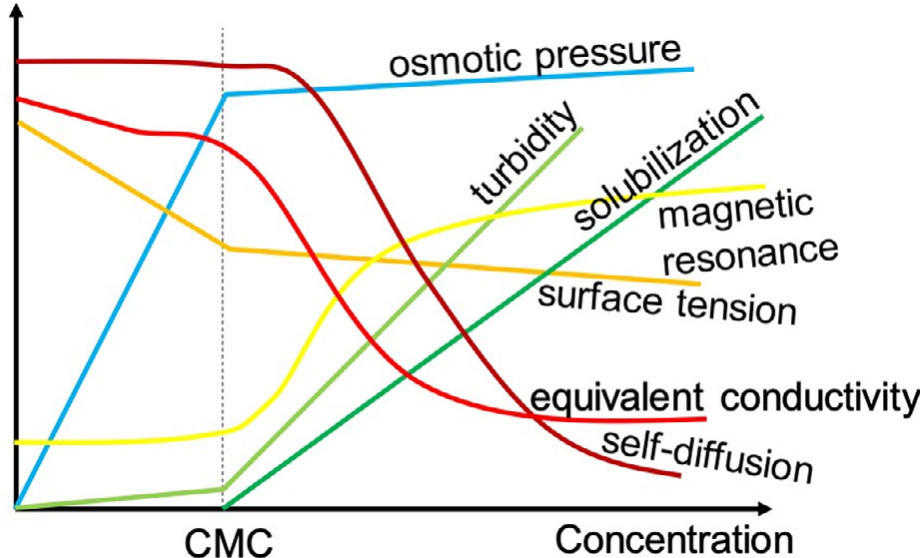
Major properties employed in the study of critical micelle concentration (adapted from Lindman and Wennerström, 2019, 1980).

A fundamental step in understanding the aggregation behaviour of surface‐active compounds is the determination of the minimal aggregation concentration, the CMC which is defined as the concentration of surface‐active compound in solution above which micelle formation takes place. For many systems, further addition of surface‐active compounds past the CMC does not induce greater phenotypic change, although in some cases secondary CMC values, above which the aggregate change in shape, size and aggregation number, are experimentally reported and theoretically described (Ruckenstein and Nagarajan, 1975; Bergström, 2015). As far as the evaluation of CMC is concerned, one can measure several physical properties (e.g. turbidity, surface tension or diffusion coefficients), qualitatively presented in Fig. 2, using various equivalent techniques (e.g. tensiometry, NMR, light scattering).

The selection of a measurable physical property is often adapted for the type of surface‐active compound; for instance, surface tension gives reliable results when studying the CMC of long‐chain amphiphiles, which generally associate faster than small‐chain ones, and ionic compounds (Prosser and Franses, 2001; Lee *et al*., 2008). However, for long equilibration processes, measurement of surface tension can be limiting due to evaporation. To overcome this problem, and when access to a tensiometer is not available, many other properties and experimental techniques offer valid results. These include the following: self‐diffusion NMR, measuring the difference in diffusion coefficient of single and aggregated surfactants, fluorescence spectroscopy, measuring the intensity ratio between the first and third vibronic peaks (I_1_/I_3_) of an internalized hydrophobic probe like pyrene, of which the emission properties are strongly affected by the medium polarity, static light scattering, measuring turbidity and so on (Fig. 2).

In terms of convenience, turbidity is highly practical because it can be measured at a fixed angle and wavelength using a common UV‐Vis spectrometer or dynamic light scattering (DLS) apparatus present in many chemistry and biology laboratories. However, turbidity is less precise than surface tension measurements. Self‐diffusion NMR is a precise technique for the spectroscopic resolution of mixtures, but experiments can be quite long at low concentrations due to the intrinsically low sensitivity of NMR. The technique also requires an NMR spectrometer and enough technical experience of the user. Measuring the spectral emission of an external probe may then be preferential as it only requires access to a spectrofluorometer or even to UV‐Vis spectrometer, two instruments widely found in many laboratories, and use of a low‐cost molecular probe like pyrene. This approach is also interesting because it provides information about the local polarity around the probe and information such as permeability to water of micellar aggregates (Basu Ray *et al*., 2006). However, as many conceptually similar methods, one should be aware the probe could potentially perturb the self‐assembly conditions. All in all, measuring CMC is quite a straightforward experiment, which can be performed in most chemistry and biology laboratories, often employing already existing instrumentation. However, surface tension and CMC do not constitute sufficient pieces of data to fully describe the aggregation behaviour of amphiphiles.

**Fig. 3:**
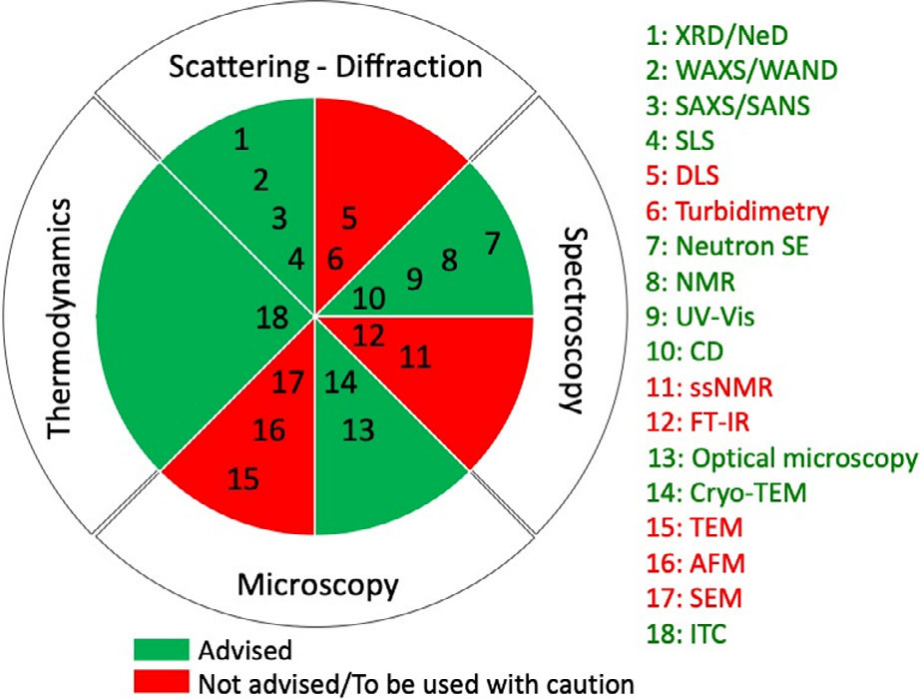
Common techniques that can be employed in the study of amphiphile self‐assembly in solution, classified in four domains. Techniques highlighted in green are the preferred techniques. Those highlighted in red, which, often requiring sample drying, do not meet the necessary standards to be safely used to study self‐assembly of amphiphiles in solution under native conditions. The techniques are classified by the type of information to which they provide access.

Evaluation of size and morphology of diluted self‐assembled amphiphiles, but also the structure and interactions of soft condensed aggregates, require a more complex analytical approach. Access to the necessary experimental tools is often limited, while data analysis and interpretation are rarely accessible to beginners. Moreover, crossing the results from at least two complementary techniques is often required. The most common techniques employed in the advanced study of the solution self‐assembly properties of surface‐active compounds can be divided into four main categories: scattering/diffraction, spectroscopy, microscopy and thermodynamics (Fig. 3). Some of the techniques depicted in Fig. 3 are strongly advised (in green); however, other ones (in red), like the popular scanning electron microscopy (SEM), have some intrinsic conditions of use, which are not generally compatible with the study of self‐assembly in solution. If, at a first glance, some of these latter technologies could provide some information, they are actually not advised or, if employed, the result should be interpreted with caution and combination with at least another more appropriate technique is necessary. Table 1 also classifies these same techniques by their functional use and provides further information with regards to: the typical size range accessible, the physical state of the sample, as well as the limitations in terms of accessibility to the equipment and complexity in terms of data treatment and interpretation. Some of these techniques have been reviewed by Yu *et al*. (2013) within the context of soft materials (Yu *et al*., 2013).

**Table 1:** Techniques employed in the characterization of self‐assembly properties in solutions of surface‐active compounds. These techniques are grouped by their functional use. Details relating to size range accessible; sample physical state; general accessibility to equipment and experimental complexity are also provided.

Information	Technique	Size domain	Physical state D: diluted C: concentrated	Level of accessibility	Level of analysis
Morphology & Size	SAXS	1–500 nm	D/C solution	Limited	Experienced
	SANS	1–500 nm	D/C solution	Very limited	Experienced
	SLS	200–1000 nm	D solution	Limited	Experienced
	Cryo‐TEM	nm to μm	D solution	Limited	Medium
	Optical microscopy	~ 200 nm–mm	D/C solution	Broad	Medium
	TEM	Å to μm	Powder	Medium	Beginner
	AFM	nm to μm	Powder	Medium	Medium
	SEM	> 100 nm	Powder	Medium	Medium
Size only	DLS	nm to ~ 1 μm	D solution	Broad	Medium
	Turbidimetry	nm to μm	D solution	Broad	Beginner
Structure	XRD/NeD	< 5 nm	D/C solution Powder	Broad/Limited	Medium
	WAXS	< 5 nm	D/C solution Powder	Limited/Very Limited	Medium
	PLM	~ 200 nm–mm	D/C solution	Broad	Medium
	CD	nm to μm	D solution	Medium	Beginner
	TEM	Å to μm	Powder	Medium	Beginner
	FTIR	< nm	Powder	Broad	Beginner
Dynamics	Neutron SE	Å to 100 nm	D/C solution	Very limited	Experienced
	NMR	< 1 nm	D solution	Broad	Experienced
Interactions	ITC	Å	D solution	Limited	Experienced
	WAXS	< 5 nm	D/C solution Powder	Limited/Very Limited	Experienced
	UV‐Vis	Å	D solution	Broad	Experienced
	CD	nm to μm	D solution	Medium	Experienced
	NMR	< nm	D solution	Broad	Experienced
	FTIR	< nm	Powder	Broad	Experienced
	ssNMR	< nm	Powder	Limited	Experienced

The most reliable way to measure size and morphology of self‐assembled aggregates in solution, and colloidal structures in general, is the combination of small‐angle X‐ray/ neutron scattering (SAXS/ SANS respectively) or static light scattering (SLS) with cryogenic transmission electron microscopy (cryo‐TEM) (Fan and Wang, 2019). SAXS/SANS generally provide a rich set of data on the aggregate structure (morphology, size, distribution of matter) for colloids of which at least one dimension is below about 300 nm. Access to SAXS instruments, although limited, is still more convenient than access to neutron facilities. However, SANS is sometimes preferred if the contrast between the electron densities of the amphiphile and the solvent is too low for X‐rays, or if X‐ray exposure degrades the sample during the experiment. SLS is generally used to probe colloids of hydrodynamic diameter above 300–500 nm.

Interestingly, SAXS employed with synchrotron radiation provides access to fast acquisition rates, meaning that many self‐assembly processes can be measured in a time‐resolved (as low as the millisecond scale) *in situ* approach (Baccile *et al*., 2016). SANS provides the so‐called ‘contrast‐matching’, which allows selective study of specific regions in supramolecular aggregates (e.g. hydrophilic headgroup, hydrophobic core in micelles, bilayers, vesicles…) with sub‐nanometre resolution, by controlling the contrast between the aggregate and the solvent. This is easily done by controlling the hydrogen‐deuterium (H/D) ratio in the solution, for instance by mixing hydrogenated and deuterated solvents (e.g. H_2_O/D_2_O). To a much lesser extent, SAXS can be employed in the same way in specific Anomalous‐SAXS (A‐SAXS) experiments. These probe the enhanced scattering of selected counterions when they are irradiated at their spectral absorption edge (Sztucki *et al*., 2011, 2012). SAXS, SANS and SLS are particularly important for their statistical relevance, illustrated by a simple calculation. A typical concentration of surfactant in solution is in the order of 0.5 wt%, which, for an average molecular weight of 500 gmol^‐1^, yields a solution of about 10^‐2^ molL^‐1^. The volume explored varies roughly between 10^‐2^ to 10^2^ mm^3^, providing a range of about 10^13^ and 10^17^ molecules. In the case that the surfactant self‐assembles into ellipsoidal nanometre‐scale micelles of classical aggregation number of 100, the number of analysed objects, of which the size and morphology is averaged at once, would vary between 10^11^ and 10^15^.

This illustrates the fact that the data collected by SAXS, SANS and SLS are extremely reliable, when the experiments and data treatment are properly done. Nonetheless, these techniques suffer from a number of drawbacks. First, they are indirect techniques, as they probe the Fourier space, thus needing model‐dependent and model‐independent analyses to extract quantitative data (Fan and Wang, 2019). For many classical situations and experienced users, this is not an issue; however, complex structures can make modelling tedious, or even not possible. At the same time, newcomers are not able to exploit and interpret the data on their own, even for the simplest systems (e.g. spherical micelles). For this reason, and to avoid misinterpretation, SAXS, SANS and SLS should always be coupled to electron and/or optical microscopy, depending on the desired scale (Fan and Wang, 2019). Finally, availability of SAXS and SANS instruments is limited and requires access to large‐scale facilities like synchrotron light and neutron sources (mandatory for SANS). Additionally, the limited number of SAXS and SLS instruments available at laboratory scale may not be powerful enough to study supramolecular systems at high dilutions (< ~ 1 wt%).

A particularly popular scattering technique employed to study colloids in solution is DLS, which has the double advantage of being accessible in many laboratories and easy to use (Hassan *et al*., 2015). DLS provides information on the hydrodynamic diameter (diameter of the colloid plus its hydration corona) and size distribution, and it is very helpful for studies on colloidal stability (Hassan *et al*., 2015). However, DLS provides no information on the morphology and is characterized by a number of possible biases (e.g. scattering from dust or aggregates), which make it very prone to misinterpretation (Hassan *et al*., 2015). Hence, DLS should be used with care and as a complement to other scattering and microscopy techniques.

Whenever possible, scattering techniques should be systematically coupled to direct observation. This is not only necessary to avoid misinterpretation but also to correctly attribute those scattering signals, which can be produced by multiple structures and which cannot unambiguously be attributed (e.g. flat lamellae vs. curved vesicular bilayers). Microscopy techniques can be divided between invasive and non‐invasive. The former modifies the concentration by drying and the latter can be performed in the parent solution. Non‐invasive microscopy can be performed from the nanometre to the millimetre scales. Cryogenic transmission electron microscopy (cryo‐TEM) has a nanometre resolution and it is a convenient technique, which preserves hydration and original concentration of the sample in its parent solvent, thus providing access to the nano‐to‐microscale morphology of the aggregate. For these reasons, it is generally preferred to conventional TEM which requires sample drying (Cui *et al*., 2007). Cryo‐TEM is nonetheless employed for diluted samples (< 1‐2 wt%) and artefacts due to vitrification, ethane adsorption and poor statistics (like any microscopy technique) could occur (Klösgen and Helfrich, 1993; Cui *et al*., 2007). For this reason, the analysis should be approached with caution, repeated, and if possible, coupled with scattering techniques. Finally, access to a cryo‐TEM microscope is limited. Standard TEM microscopes can be employed under cryogenic conditions; however, only a reduced number of laboratories are actually equipped and possess the know‐how to perform routine cryo‐TEM experiments.

Optical microscopy, despite its lower resolution (micrometre to millimetre scales), can be used in a hydrated environment, constitutes the alternative to cryo‐TEM for larger samples (micron‐scale) and can be coupled to data obtained by SLS. Any microscopy approach requiring drying (standard TEM, SEM or even atomic force microscopy, AFM) should be avoided or at least employed with extreme care. This is due to the amphiphile concentration changes and aggregation or unexpected phase transitions, not reflecting the self‐assembled state in solution which can potentially occur. For a robust interpretation, combining SAXS/SANS and cryo‐TEM data should be coherent and provide the same information.

The structure of the condensed state (crystalline or liquid crystalline) of self‐assembled aggregates formed by surface‐active compounds can be accessed with diffraction techniques and polarized light microscopy (PLM) (Stribeck, 2007). X‐ray diffraction (XRD), and in rare cases neutron diffraction (NeD), is very practical, although standard diffractometers in θ‐2 θ geometry do not allow a reliable analysis of wet samples and diluted solutions. In this case, one should employ a wide‐angle X‐ray scattering (WAXS) configuration. This may sometimes be available as a supplementary tool in a SAXS instrument, at a laboratory scale or at a synchrotron facility. Similar to scattering techniques, XRD or WAXS, are measured in the Fourier space but in this case, modelling is not necessary and data interpretation generally occurs on the simple analysis of the peak positions relative one to the other and the general principles of crystallography apply. In practice, the variety of possible crystal systems is generally limited to few recurrent ones (2D oblique lamellar, 3D hexagonal and cubic) compared with crystalline inorganic solids and straightforward interpretations are not uncommon.

Neutron diffraction could replace use of X‐rays in specific cases of sample instability under X‐rays, seeking light‐weight atoms like hydrogen, or contrast‐matching experiments. PLM has long been used to analyse surfactant mesophases, however despite the ease of accessibility to polarized light microscopes, image analysis requires a long experience in the field and coupling to diffraction experiments may be necessary (Lee *et al*., 2018). Circular dichroism (CD) is a spectroscopic technique, which is useful to probe chirality in molecular and supramolecular systems in solution. Finally, same as above, any technique which requires sample drying like infrared spectroscopy, FTIR, and standard TEM should be employed with care.

More advanced studies on the functionality of surface‐active compounds may include analysis of the dynamics and intra/intermolecular interactions in the aggregates formed by these compounds. Probing the elastic constants of lipid membranes ($k_{c}$, ${\overset{‐}{k}}_{c}$), the thermodynamic and kinetic parameters of self‐assembly but also the possible existence of raft regions in lipid membranes are hot topics in biophysics and quantification of intermolecular forces are all important aspects of advanced characterization of self‐assembled systems (Helfrich, 1978; McIntosh and Simon, 1996; Holmberg *et al*., 2002; Loh *et al*., 2016; Monzel and Sengupta, 2016; Sezgin *et al*., 2017). Accessing this class of information requires experience, even if the analytical technique itself is relatively accessible and easy to use. The study of both local (e.g. intra‐aggregate) and collective (e.g. membrane fluctuations) dynamics of self‐assembled systems can be performed with neutron spin echo (NSE) or NMR spectroscopy (Detail of both these techniques is provided in Table 1) (Tiddy, 1972; Brown and Schofield, 1975; Villeneuve *et al*., 2006; Mell *et al*., 2013; Monzel and Sengupta, 2016). Details of both these techniques are summarized in Table 1, and Monzel and Sengupta (2016) provides further detail of these techniques space and time scales as well as their advantages and inconveniences (Monzel and Sengupta, 2016).

Self‐surfactant‐solvent and intersurfactant interactions can be explored with isothermal titration calorimetry (ITC) (Loh *et al*., 2016). This is the preferred technique for quantifying the enthalpy change, association constant (or binding affinity) and stoichiometry, and consequently the Gibbs free energy and entropy changes, between two or more molecules in solution (Kayitmazer, 2017). However, ITC apparatus is not readily accessible, and its usage requires a high level of experimental practice and data analysis. Collective intermolecular forces can be quantified in so‐called pressure‐distance experiments, consisting in following a structural parameter (e.g. interlamellar distance in lamellar phases) with osmotic pressure. These experiments can be performed with a more accessible X‐ray (or neutron, in some cases) diffraction apparatus. Despite the experimental ease, the understanding and analytical treatment of data related to intermolecular forces is still quite complex and reserved to expert users (Leneveu *et al*., 1976; Dubois *et al*., 1998; Parsegian and Zemb, 2011). Spectroscopic techniques like UV‐Vis, FTIR, CD and NMR can also provide a qualitative insight on interactions (Table 1). NMR is of particular interest for the broad panel of 2D and 3D homo and heteronuclear experiments based on intramolecular, through‐bond, *J*‐couplings as well as intermolecular, through‐space, dipolar and quadrupolar couplings. Solution NMR efficiency may sometimes be reduced due to short relaxation times, (such as in the case of large, slow‐tumbling, aggregates), and use of solid state NMR (ssNMR) may be necessary. However, ssNMR generally requires sample dehydration, which can modify the structure and interactions, thus leading to misinterpreted data. Using ssNMR with wet samples should be utilized when possible (Nonappa and Kolehmainen, 2016).

## Conclusion

A major issue facing the field of microbial surface‐active compound research has been that the discovery and/or biotechnological application of new biosurfactant‐producing organisms has been seen as an easy opportunity for a research project and subsequent publication. This has led to a multitude of papers in the literature that are not only of little scientific value but are also misleading through repeated citation. In this paper, we have critically evaluated a number of protocols and methodologies that have been utilized by various studies ranging in topic from the initial reporting of microbial surface‐active compound production to process development for the exploitation of these compounds for industrial application and characterization of their functionality. Within each section of the paper, we have advised on which techniques are favourable and which techniques should be avoided. Additionally, we have advised upon a number of ‘gold standard’ techniques and experimental evidence that should be both employed and provided in publications resulting from future studies relating to microbially produced surface‐active compound studies. These techniques and experimental evidence we have judged to be required, desirable and unreliable are summarized in Table 2. We hope that the views and guidelines expressed in this paper regarding the use of stringent protocols will lead to a stricter approach in the process of carrying out research and future reporting relating to microbial surface‐active compounds. Studies that do not utilize the necessary techniques and equipment to carry out a reliable, accurate examples of work describing, and characterizing production of surface‐active compounds should not be published while making unsupported claims.

**Table 2:** A list summarizing both techniques and experimental evidence employed and generated in the field of microbial surface‐active compound research. The list is divided into the same four broad subsections of research discussed in this paper. Techniques and experimental evidence for publication initial biosurfactant characterization, strain identification and process development have been listed as ‘essential’, ‘desirable’ and ‘not‐recommended’. Techniques employed in functionality testing are grouped as ‘recommended’ and ‘not‐recommended’.

Protocol/Technique	Importance
Initial biosurfactant characterization	
Basic Identification techniques (e.g. Drop Collapsing test etc.)	Not‐Recommended
End point phenotypic analysis (e.g. surface tension, emulsification, oil dispersion)	Desirable^a^
Phenotypic analysis throughout growth cycle (e.g. surface tension, emulsification, foam formation)	Essential
Victoria Pure Blue BO microtitre plate assay	Desirable with caution^b^
Cetyltrimethylammonium bromide (CTAB) assay	Not‐Recommended
Utilization and description of biosurfactant extraction and purification methodologies	Essential
Calculation of approximate production amounts/ titres	Essential
Colorimetric Analysis (e.g. Orcinol Assay)	Not‐Recommended
Thin‐layer Chromatography	Not‐Recommended
Fourier Transform Infrared Spectroscopy (FTIR)	Not‐Recommended
High Performance Liquid Chromatography‐Mass Spectroscopy (HPLC‐MS)	Essential
UPLC‐MS/MS	Desirable
Nuclear Magnetic Resonance (MNR) Spectroscopy	Desirable
Biosynthesis pathway elucidation	Essential
Strain identification	
Colony morphology	Not‐Recommended
Cell morphology/staining	Not‐Recommended
Biochemical Testing	Not‐Recommended
Phylogenetic analysis using reference genes (e.g. 16S rRNA)	Essential
Whole genome sequencing	Desirable^c^
Process development	
Generation of highly purified standards of the product(s)	Essential
Accurate assessment of congener ratios in the product(s)	Essential
Separate standards for the most abundant congeners	Desirable
Generation/ purchase of standards for all substrates using in the production process	Essential
Accurate determination of product concentrations/ titres (g l^‐1^), productivities (gl h^‐1^), yields (on substrate (%)) and recovery yields (%) (of purification process)	Essential
Determination and reporting of potential contaminants. (e.g. Fatty acids, carbohydrates, proteins and endotoxins)	Essential
Determination and reporting of any contaminating DNA within the product following the production process	Desirable
Correct usage of reporting terminology (i.e. yield, titre, purity, uniformity, etc.)	Essential
Functional characterization	
Small‐angle X‐ray scattering (SAXS)	Essential
Small‐angle neutron scattering (SANS)	Recommended
Static light scattering (SLS)	Recommended
Cryogenic transmission electron microscopy (Cryo‐TEM)	Essential
Optical microscopy	Recommended
Transmission electron microscopy (TEM)	Not‐Recommended
Atomic force microscopy (AFM)	Not‐Recommended
Scanning electron microscopy (SEM)	Not‐Recommended
Dynamic light scattering (DLS)	Not‐Recommended with Caution^d^
Turbidimetry	Not‐Recommended
X‐ray diffraction (XRD)/ Neutron diffraction (NeD)	Recommended
Wide‐angle X‐ray scattering (WAXS)	Recommended
Polarized light microscopy (PLM)	Recommended
Circular dichroism (CD)	Recommended
Fourier Transform Infrared Spectroscopy (FTIR)	Not‐Recommended
Neutron Spin Eco (SE)	Recommended
Nuclear Magnetic Resonance (MNR) Spectroscopy	Recommended
Isothermal titration calorimetry (ITC)	Recommended
Ultraviolet visible spectroscopy (UV‐Vis)	Recommended
Solid state nuclear magnetic resonance (ssNMR)	Not‐Recommended

^a^ Only recommendable for the high‐throughput screening of a large number of samples.

^b^ Relatively new assay format and may require further validation by independent research teams.

^c^ WGS can be utilized when strain identification via reference gene sequencing is inconclusive.

^d^ DLS is not recommended, but it is in practice widespread, highly used and often unavoidable. Data to be interpreted with caution.

## Conflict of interest

None declared.
